# Spring phenology of cotton bollworm affects wheat yield

**DOI:** 10.1002/ece3.2719

**Published:** 2017-01-23

**Authors:** Jian Huang, Jing Li

**Affiliations:** ^1^ Institute of Desert and MeteorologyChina Meteorological AdministrationUrumqiChina; ^2^ Central Asian Research Center of Atmospheric SciencesUrumqiChina; ^3^ Xinjiang Plant Protection StationUrumqiChina

**Keywords:** abrupt climate change, climate change, *Helicoverpa armigera*, spring phenology

## Abstract

Climate change has changed numerous species phenologies. Understanding the asynchronous responses between pest insects and host plants to climate change is helpful in improving integrated pest management. It is necessary to use long‐term data to analyze the effects of climate change on cotton bollworm and wheat anthesis. Data for cotton bollworm, wheat yield, and wheat anthesis collected since 1990 were analyzed using linear regression and partial least‐squares regression, as well as the Mann–Kendall test. The results showed that warmer temperatures in the spring advanced the phenologies of cotton bollworm and wheat anthesis, but the phenology changes in overwintering cotton bollworm were faster than those in wheat anthesis, and the eclosion period of overwintering was prolonged, resulting in an increase in overwintering adult abundance. This might lead to more first‐generation larvae and subsequent wheat damage. An early or late first‐appearance date significantly affected the eclosion days. The abrupt changes of phenologies in cotton bollworm, wheat anthesis, and climate were asynchronous, but the abrupt phenology changes occurred after or around the climate abrupt change, especially after or around the abrupt changes of temperature in March and April. The expansion of asynchronous responses in the change rate of wheat anthesis and overwintering cotton bollworm would likely decrease wheat yield due to climate warming in the future. Accumulated temperature was the major affecting factor on the first eclosion date (*t*_1_), adult abundance, and eclosion days. Temperatures in March and April and precipitation in the winter mainly affected the prepeak date (*t*_2_), peak date (*t*_3_), and postpeak date (*t*_4_), respectively, and these factors indirectly affected wheat yield. Thus, the change in the spring phenology of the cotton bollworm and wheat anthesis, and hence wheat yield, was affected by climate warming.

## 1 Introduction

It is challenging for scientists to demonstrate how climate impacts natural ecosystems (Parmesan & Yohe, 2003; Root et al., 2003; Stenseth et al., 2002). The literature shows that climate warming changes the phenologies of terrestrial organisms (Parmesan, 2007; Satake, Ohgushi, Urano, & Uehimura, 2006; Westgarth‐Smith, Leroy, Collins, & Harrington, 2007) and crop yields (Huang, 2016; Huang & Ji, 2015; Wang et al., 2008). Different trophic levels have different temperature sensitivities (Berggren, Björkman, Bylund, & Ayres, 2009; Voigt et al., 2003), and insects usually show more robust responses to climate change than do plants (Gordo & Sanz, 2005; Parmesan, 2007). This is likely because insect metabolism is more sensitive to increases in temperature than plant metabolism (Bale et al., 2002; Berggren et al., 2009). These different responses to climate change produce matched or mismatched phenologies (Anderson, Gurarie, Bracis, Burke, & Laidre, 2013; Cushing, 1969). An improved understanding of how climate impacts ecological processes and the involved mechanisms would result in better predictions of the effects of future climate change.

The match/mismatch hypothesis illustrates recruitment variation in a population via the relationships between the phenological changes of different trophic levels (Durant, Hjermann, Ottersen, & Stenseth, 2007). If there is a match between requirement and availability, recruitment increases; if there is a mismatch, there are lower survival and recruitment (Durant et al., 2007). Mismatches of phenologies can result in severe harmful impacts on organisms that feed on ephemeral resources, especially larvae that occur before the host plant budburst (Bale et al., 2002). An advance or a delay of host plant phenology can cause vulnerabilities to both species because this can adversely affect fitness (Singer & Parmesan, 2010).

In temperate ecosystems, many species begin to acquire food and develop in the spring. However, spring weather has been significantly changed by climate change (Schwartz, Ault, & Betancourt, 2013), which has led to numerous organisms responding to warmer temperatures in the spring (Hughes, 2000; Wuethrich, 2000) via the advancement or delay in their phenologies, resulting in subsequent further resource exploitation (Leinonen & Hänninen, 2002). As a physiological restriction and as a cue that sets the biological clock, temperature directly determines the timing of phenology (Ausín, Alonso‐Blanco, & Martínez‐Zapater, 2005; Gwinner, 1996). Insects are particularly sensitive to temperature changes (Deutsch, Tewksbury, Huey, Sheldon, & Ghalambor, 2008). Temperature change can significantly affect the survival and development of cotton bollworm (Wu & Guo, 2005), and the eclosion of cotton bollworm is advanced in warmer temperatures (Huang & Li, 2015; Ouyang et al., 2016).

The cotton bollworm, *Helicoverpa armigera* (Hübner) (Lepidoptera, Noctuidae), is one of the most serious insect pests around the globe (Fitt, 1989; Zalucki, Daglish, Firempong, & Twine, 1986; Zalucki et al., 1994) and is characterized by its high fecundity, polyphagy, facultative diapause, and high mobility (Wu & Guo, 2005). In northern China, it usually produces four (Ge, Chen, Parajulee, & Yardim, 2005) or five generations (Wu & Guo, 2005) in a year. In the temperate region, diapausing pupae can successfully live through the winter (Wu & Guo, 2005). After emerging from overwintering pupae, the adults feed on wheat pollen, the first‐generation larvae feed on wheat, and other three generations mainly feed on corn and cotton (Wu & Guo, 2005). Changes in agricultural intensification and climate can cause great fluctuations in the cotton bollworm population (Ouyang et al., 2014), but few outbreaks have appeared in the past 20 years due to the wide adoption of transgenic crops (Wu, Lu, Feng, Jiang, & Zhao, 2008).

Although the warmer temperatures can increase the survival rate of overwintering cotton bollworm pupae (Huang & Li, 2015; Liu, Gong, et al., 2007; Liu, Zheng, Zhao, & Chen, 2007; Liu et al., 2009; Ouyang et al., 2016), adult moths in the spring are likely to face a scarcity of diet because wheat anthesis and pupae might have different responses to the increase in winter temperatures (Ge et al., 2005; Reddy et al., 2015). Therefore, the increase in winter temperature might lead to a phenological mismatch between wheat anthesis and *H. armigera* eclosion. Wang et al. (2008) stated that wheat anthesis advanced by 0.48 days per year. Huang and Li (2015) and Ouyang et al. (2016) illustrated the phenology response of *H. armigera* to climate change. However, few studies have examined the relationship between the phenology of *H. armigera* and wheat anthesis. In addition, how the phenology of *H. armigera* in the spring affects wheat yield is not clear.

Understanding asynchronous responses between pest insects and host plants to climate change is helpful for improving integrated pest management. Therefore, we explored how changes in *H. armigera* abundance and phenology and wheat anthesis from climate warming affect wheat yield. Thus, the objectives of this study were as follows: (1) detect the phenological trends of overwintering *H. armigera* adults and wheat anthesis; (2) analyze the change trend of wheat yield; (3) explore the relationship between the phenology of *H. armigera* and wheat anthesis and yield; and (4) determine which climatic factors may significantly affect the phenology of *H. armigera*, wheat anthesis, and wheat yield.

## 2 Materials and Methods

The study area is in Maigaiti County (38°25′–39°22′N, 77°28′–79°05′E, altitude of 1,155–1,195 m), which covers a total area of 11,023 km^2^ and is located in the western Tarim Basin, Xinjiang Uygur Autonomous Region, China (Zhang & Zhang, 2006). Cotton, wheat, and corn are the major crops in this region. Adult moths were captured using a blacklight lamp (20 W) at night from early April to late September since 1990. The blacklight lamp, placed in an open field, was turned on after sundown and turned off after sunrise every day. The all setting criteria met state rules. For climate conditions in this region, detailed data, and experimental methods for *H. armigera,* see Huang and Li (2015). Climate data, which included the mean temperature (*T*_mean_), maximum temperature (*T*_max_), minimum temperature (*T*_min_), and precipitation and wheat data were observed by the Maigaiti Meteorology Administration. The collection of all climate data followed the “China Agricultural Meteorological Observation Guidelines” (Xu & She, 1980).

To avoid any potential micrometeorological effects, the investigated wheat‐planted area of 2 ha in Maigaiti was approximately 100 m away from any highway, building, or river. The wheat area was quartered into four replicates. Wheat phenologies were observed on 30 individual plants in an area of 2 m^2^ for every plot. The phenology observation region was tagged, and all observations and measurements were conducted on plants in the tagged areas. Observations of the plants were conducted every 2 days during the whole growing period. When 50% of the wheat plants in the tagged area changed developmental stages, the calendar dates were recorded. Every growing stage was confirmed according to the Zadoks scale (Zadoks, Chang, & Konzak, 1974). During the study period, the observation plots were changed every year, but they were always located within 100 m of the weather station.

Climate data have been recorded since 1960, and wheat phenological and yield data have been recorded since 1983; experimental data for *H. armigera* were recorded from 1990 to 2007. For comparison purposes, the *H. armigera*, climate, and wheat data from the period of 1990–2007 were used. To illustrate the effects of climate change on the spring phenology of *H. armigera* and wheat anthesis, the accumulated temperature (AT) and *T*_mean_ were calculated from January 1 to each eclosion period of overwintering *H. armigera* and wheat anthesis. Ouyang et al. (2016) defined the eclosion period of the overwintering generation of *H. armigera* as the first eclosion date (*t*_1_), prepeak date (*t*_2_), peak date (*t*_3_), postpeak date (*t*_4_), and the last eclosion date (*t*_5_). Thus, AT_1–5_ represents the AT from January 1 to *t*_1–5_, respectively. To conveniently study the interval days between appearance dates (*t*_1_–*t*_5_) and wheat anthesis, gap_1_, gap_2_, gap_3_, gap_4_, and gap_5_ were used for the interval days between the appearance dates of *t*_1_, *t*_2_, *t*_3_, *t*_4_, *t*_5_, and wheat anthesis, respectively. The Mann–Kendall test was used to identify abrupt changes in phenology and climate.

Linear regression was used to analyze the trends over time. The relationships between the phenologies of cotton bollworm and wheat anthesis and climate date were determined with regression functions and Pearson's correlation analysis. To avoid multicollinearity, a common phenomenon in multivariate analysis, a partial least‐squares (PLS) regression was employed to determine the relative impact (explained variance) of the factors, such as the relative impacts of temperature, precipitation, days, and abundance. All analyses were conducted with SPSS 17.0.

## 3 Results

### 3.1 Relationships between the appearance dates of the overwintering generation and the anthesis and yield of wheat

**Figure 1:**
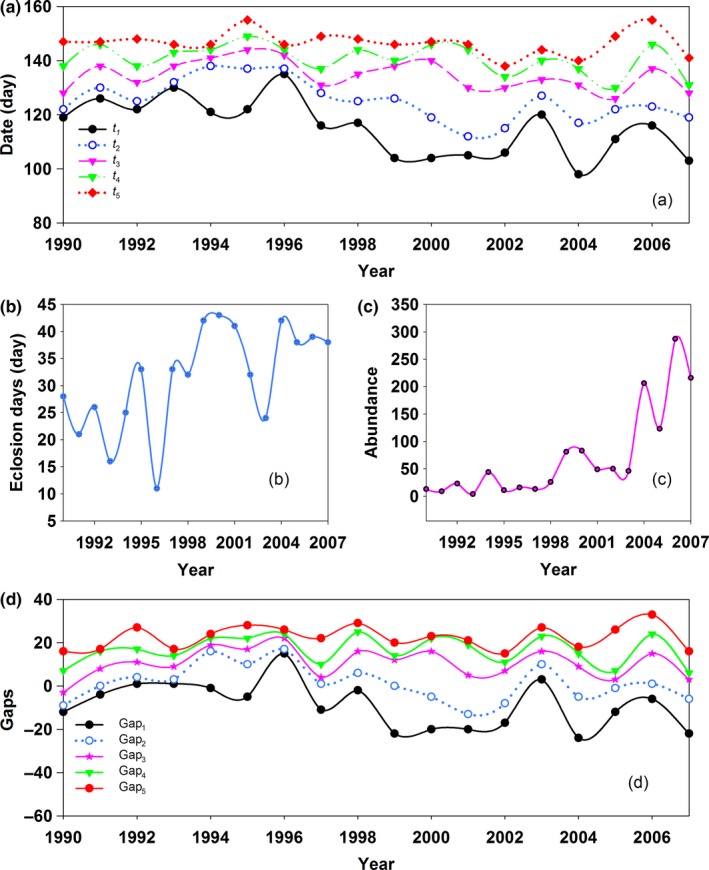
Appearance dates (a), eclosion days (b), and abundance (c) of overwintering generation cotton bollworm, and gaps between wheat anthesis and the dates of *t*_1_–*t*_5_ (d)

The phenology of wheat anthesis and the dates of *t*_1_–*t*_5_ of overwintering cotton bollworm showed advanced trends, and *t*_1_ and *t*_2_ had significant correlations with time (Table 1). The ranges of the dates of *t*_1_–*t*_5_ were 37, 23, 24, 25, and 17 days (Figure 1a), respectively. The standard deviations (*SD*) of *t*_1_–*t*_5_ were 10.2831, 7.5581, 5.3821, 5.4139, and 4.3008 days, respectively. This suggested that the date of *t*_1_ had the largest fluctuation range among the dates and was the most easily affected date by climate change. The date of *t*_1_ was advanced by 1.276 days per year with climate warming (Table 1). The eclosion days of the overwintering generation were prolonged by 1.09 days per year (Table 1). However, the eclosion days varied from 11 to 43 days (Figure 1b) with an *SD* of 9.3433 days, which approximated the *SD* of *t*_1_; this might suggest that eclosion days and the date of *t*_1_ have a similar variation trend. Furthermore, the proportion of explained variance of *t*_1_–*t*_5_ to eclosion days was 57.8%, 35.7%, 5.9%, 0.3%, and 0.2% using PLS analysis, respectively. This suggested that, in terms of *t*_1_–*t*_5_, the date of *t*_1_ had the greatest effect on eclosion days.

**Table 1:** Trends over time in appearance dates, abundance of cotton bollworm and anthesis and yield of wheat from 1990 to 2007

Parameter		Linear regression	*R* ^2^	*p*	Trend
*t*_1_	The first eclosion date	*Y* = −1.277*X* + 127.405	.439	.003	Advanced
*t*_2_	Prepeak date	*Y* = −0.788*X* + 132.712	.310	.016	Advanced
*t*_3_	Peak date	*Y* = −0.382*X* + 138.183	.143	.121	Advanced
*t*_4_	Postpeak date	*Y* = −0.420X + 144.601	.172	.087	Advanced
*t*_5_	The last eclosion date	*Y* = −0.193*X* + 148.373	.053	.357	Advanced
2δ	Eclosion days	*Y* = 1.090*X* + 20.980	.384	.006	Prolonged
A	Abundance	*Y* = 12.244*X* − 44.092	.620	<.001	Increased
W	Wheat yield	*Y* = 201.363*X* + 2838.3	.967	<.001	Increased
F	Wheat anthesis	*Y* = −0.333*X* + 127.22	.218	.051	Advanced

*Y*, parameters in population dynamics of overwintering generation; *X*, year from 1990 to 2007.

**Figure 2:**
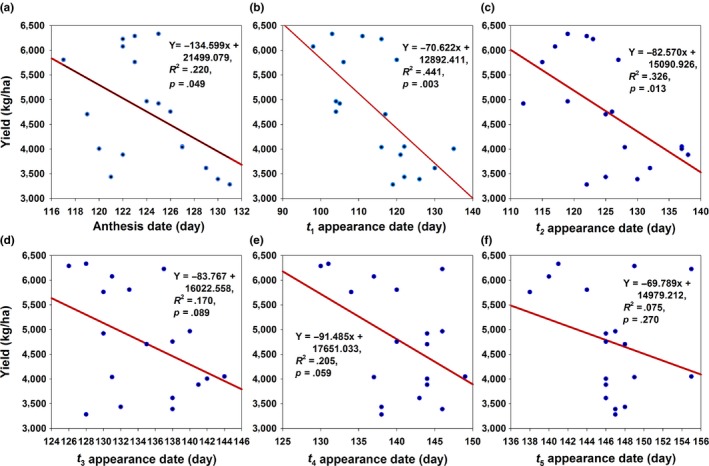
Relationships between wheat yield and anthesis and the dates of *t*_1_–*t*_5_

Population abundance of the overwintering generation increased by 12.444 per year, and wheat yield increased by 201.363 kg/ha per year (Table 1). Population abundance varied from 7 to 287 (Figure 1c), and wheat yield varied from 2,280 to 6,389 kg/ha. In addition, wheat anthesis advanced by 0.333 days per year (Table 1). The wheat yield increased by 134.599, 70.622, 82.570, 83.767, 91.485, and 69.789 kg/ha with an advanced day of wheat anthesis and *t*_1_–*t*_5_, respectively (Figure 2a–f). However, a significant correlation only existed between wheat yield and anthesis and *t*_1_ and *t*_2_ (Figure 2a–c). Among the dates of *t*_1_–*t*_5_, the advance of the *t*_4_ date had the greatest effect on wheat yield (Figure 2e), where with a 1 day advance of the *t*_4_ date, wheat yield increased by 91.485 kg/ha. This illustrated that the phenology changes of cotton bollworm were faster than those of wheat anthesis.

### 3.2 Meteorological factors affected phenology and abundance of cotton bollworm and wheat yield

**Figure 3:**
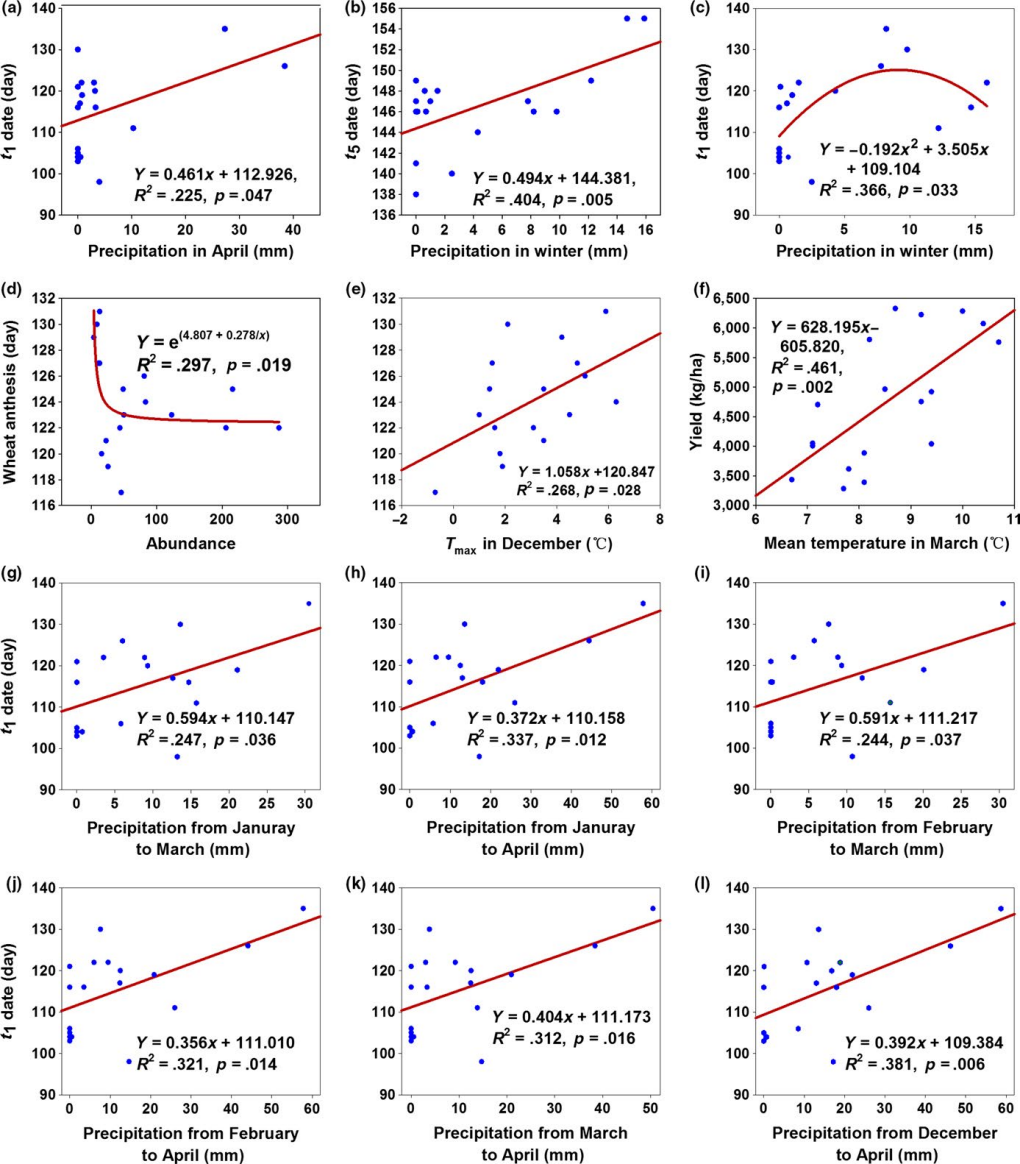
Relationships between precipitation and *t*_1_ (a, c, g–l) and *t*_5_ (b), between yield and *T*_mean_ in March (f), wheat anthesis and abundance (d), wheat anthesis and *T*_max_ in December (e)

With a 1‐mm precipitation increase in April and the winter, the dates of *t*_1_ and *t*_5_ were delayed by 0.461 and 0.494 days, respectively (Figure 3a,b), while the precipitation in April and the winter delayed the dates of *t*_2_–*t*_4_, although no significant correlations were observed (data not shown). These results showed that precipitation in April and the winter could delay the first‐appearance and end‐appearance day of overwintering pupae eclosion. However, precipitation in the winter was approximately 8 mm, and the *t*_1_ date approached the highest value (viz.) on the latest day (Figure 3c). If the linear model was selected, the relationship was insignificant (*p *=* *.058), although it showed a delayed trend. Therefore, the quadratic model was selected in this study. The combination of precipitation in different months showed significant correlations with date *t*_1_ only, and a 1 mm increase in precipitation delayed *t*_1_ 0.356–0.594 days (Figure 3g–l). The combination of precipitation in different months also delayed the dates of *t*_2_–*t*_5_, though insignificant (data not shown). These results suggested that precipitation could delay the eclosion of *H. armigera*, and the effect of precipitation from January to March on the date of *t*_1_ was the largest (Figure 3g).

**Figure 4:**
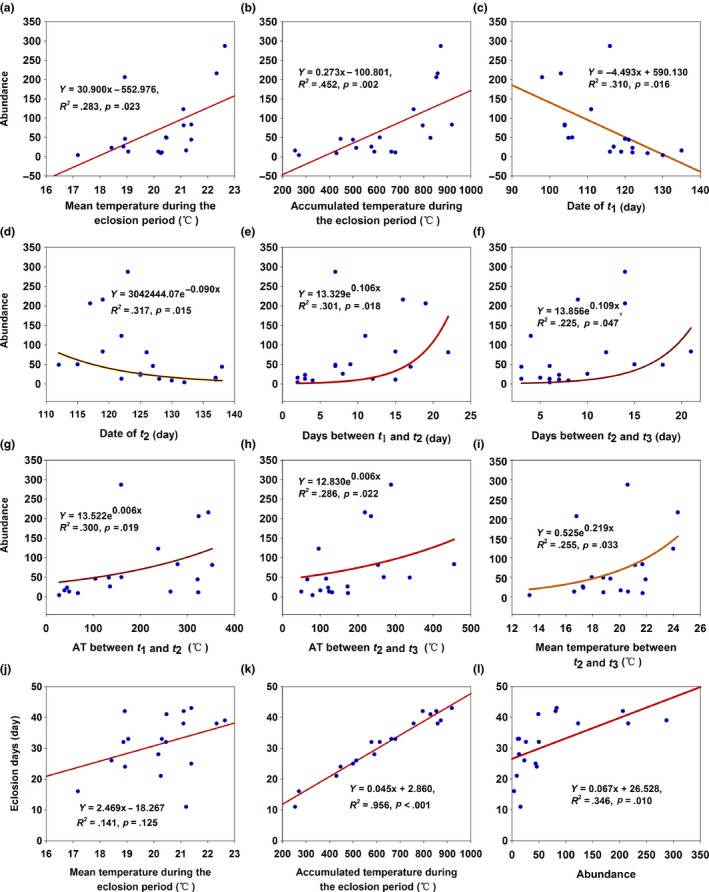
Relationships between abundance, eclosion days, *T*_mean,_ and accumulated temperature during the eclosion period

For a 1°C increase in the mean air temperature (*T*_mean_) in March, wheat yield increased by 628.195 kg/ha (Figure 3f). This suggested that precipitation delayed the appearance date of the overwintering cotton bollworm. The advance of wheat anthesis was accompanied with a greater abundance of *H. armigera* (Figure 3d), and the increase in the maximum air temperature (*T*_max_) in December delayed the date of wheat anthesis (Figure 3e). For a 1°C increase in the *T*_mean_ and AT during the eclosion period, the abundance increased by 30.900 and 0.273, respectively (Figure 4a,b), and the eclosion days increased by 2.469 and 0.045 days, respectively (Figure 4k,l). This suggested that the increase in air temperature increased the abundance and prolonged the eclosion period of overwintering cotton bollworm. However, the abundance decreased with the delay of the dates of *t*_1_ and *t*_2_ (Figure 4c,d) and increased with the increase in days between *t*_1_ and *t*_2_ (Figure 4e), days between *t*_2_ and *t*_3_ (Figure 4f), AT between *t*_1_ and *t*_2_ (Figure 4g), AT between *t*_2_ and *t*_3_ (Figure 4h), *T*_mean_ between *t*_2_ and *t*_3_ (Figure 4i), and eclosion days (Figure 4l). This suggested that abundance was affected by many factors. For a 1 increase in abundance, the eclosion period increased by 0.067 days (Figure 4l). For a 1‐day increase in eclosion period, the abundance increased by 5.195 (*Y* = 5.195*X* − 90.558, *R* ^2^ = .346, *p *=* *.010).

The *T*_mean_ in March and April advanced the dates of *t*_1_–*t*_5_ and showed significant correlations, except for the relationship between *t*_5_ and the *T*_mean_ in April; the effects of the *T*_mean_ in March on the appearance dates of *t*_1_–*t*_5_ were greater than those in April (Table 2). The advances of *t*_1_ and *t*_5_ were the greatest and the smallest, respectively (Table 2). This suggested that climate warming had the greatest impact on the appearance date of *t*_1_ and had the least impact on the appearance date of *t*_5_ because the *T*_mean_ in March and April increased by 0.146 and 0.110°C/year from 1990 to 2007 (*Y*_March_ = 0.146*x* + 7.137, *R* ^2^ = .438, *p *=* *.003; *Y*_April_ = 0.110*x* + 14.969, *R* ^2^ = .168, *p *=* *.092), respectively.

**Table 2:** Effects of mean temperatures in March and April on appearance dates, abundance, eclosion days, and wheat yield

Parameter		Linear regression	*R* ^2^	*p*	Trend
*t*_1_	The first eclosion date	*Y* = −6.356*X*_1_ + 169.480	.533	.001	Advanced
		*Y* = −3.637*X*_2_ + 173.537	.259	.031	Advanced
*t*_2_	Prepeak date	*Y* = −4.074*X*_1_ + 159.961	.405	.005	Advanced
		*Y* = −2.736*X*_2_ + 169.044	.271	.027	Advanced
*t*_3_	Peak date	*Y* = −2.293*X*_1_ + 154.109	.253	.033	Advanced
		*Y* = −1.869*X*_2_ + 164.493	.250	.035	Advanced
*t*_4_	Postpeak date	*Y* = −2.328*X*_1_ + 160.462	.258	.031	Advanced
		*Y* = −1.770*X*_2_ + 168.963	.221	.049	Advanced
*t*_5_	The last eclosion date	*Y* = −1.525*X*_1_ + 159.564	.176	.084	Advanced
		*Y* = −0.826*X*_2_ + 159.787	.076	.267	Advanced
A	Abundance	*Y* = 35.638*X*_1_ − 231.689	.257	.032	Increased
		*Y* = 23.025*X*_2_ − 296.561	.159	.101	Increased
2δ	Eclosion days	*Y* = 4.775*X*_1_ − 9.383	.360	.008	Prolonged
		*Y* = 2.851*X*_2_ − 14.325	.191	.070	Prolonged
W	Wheat yield	*Y* = 628.195*X*_1_ − 605.820	.461	.002	Increased
		*Y* = 270.827*X*_2_ + 413.551	.127	.147	Increased

*X*_1_ and *X*_2_ represented *T*_mean_ in March and April, respectively; *Y* parameters in population dynamics of overwintering generation and wheat yield.

### 3.3 Mann–Kendall tests for trends

**Figure 5:**
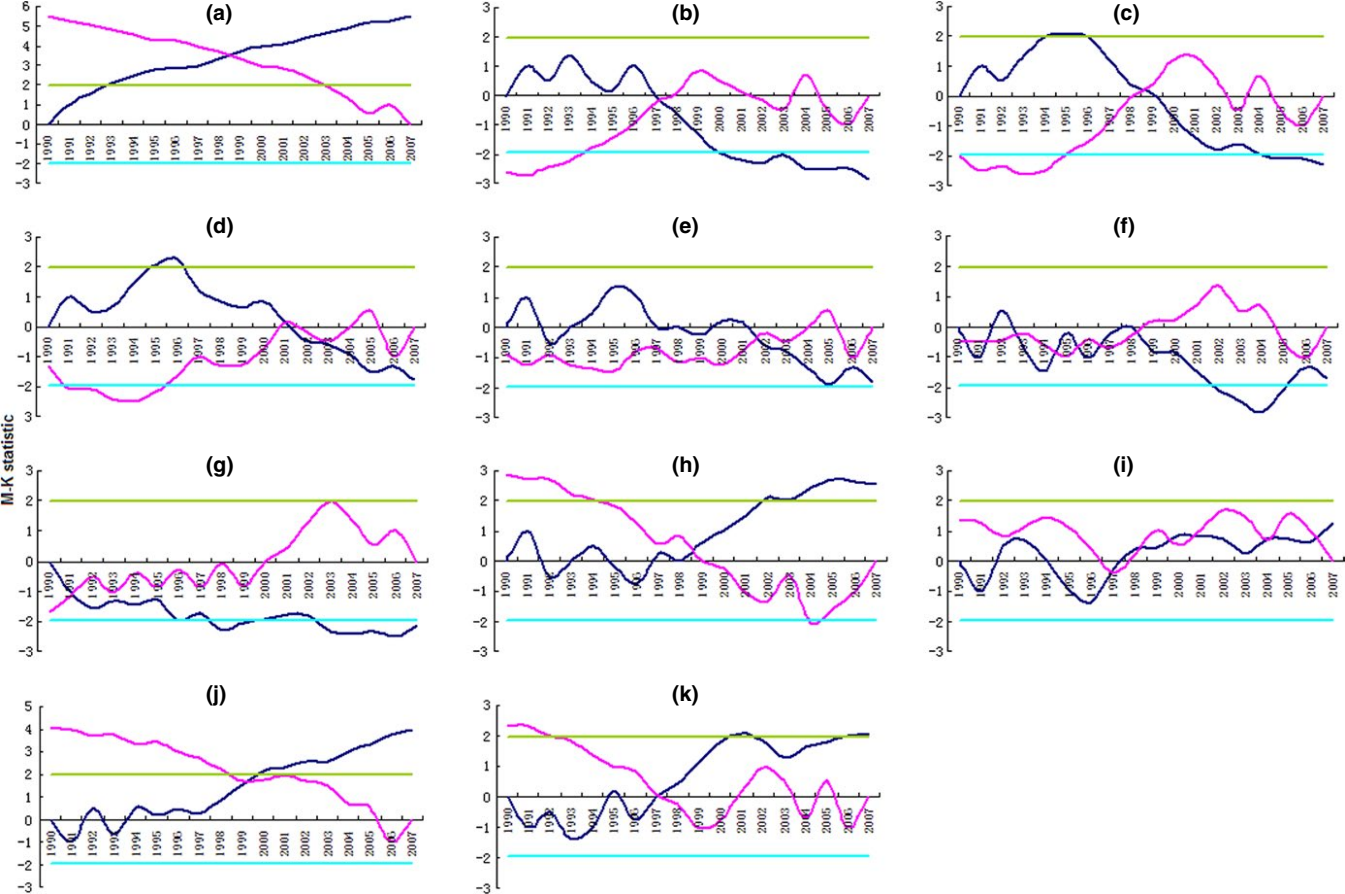
Mann–Kendall tests for wheat yield (a), date of *t*_1_ (b), date of *t*_2_ (c), date of *t*_3_ (d), date of *t*_4_ (e), date of *t*_5_ (f), anthesis (g), *T*_mean_ in March (h),*T*_mean_ in April (i), abundance (j), and eclosion period (k)

To make sure the trends of the abrupt changes of the phenologies of overwintering cotton bollworm and wheat, wheat yield, *T*_mean_ in March and April, abundance, and eclosion days, a Mann–Kendall test at a 5% significance level was employed. The results showed that the date of *t*_1_ (Figure 5b), date of *t*_2_ (Figure 5c), date of *t*_3_ (Figure 5d), date of *t*_4_ (Figure 5e), date of *t*_5_ (Figure 5f), anthesis (Figure 5g), *T*_mean_ in March (Figure 5h), abundance (Figure 5j), and eclosion period (Figure 5k) all appeared significant abrupt changes in 1997, 1999, 2001, 2002, 1998, 1991, 1999, 1999, and 1997, respectively (Figure 5b–h). Among the abrupt change years of *t*_1_–*t*_5_, the earliest and latest abrupt changes were in 1997 for *t*_1_ and 2002 for *t*_4_ (Figure 5b,e). The abrupt change years of *t*_1_ and *t*_5_ were earlier than those of *T*_mean_ in March; however, the abrupt change year of *t*_1_ was coinstantaneous with the abrupt change of *T*_mean_ in April, and the abrupt change year of *t*_5_ was later than the abrupt change of the *T*_mean_ in April. Meanwhile, the abrupt change years of *t*_2_–*t*_4_ were later than those of the *T*_mean_ in March and April (Figure 5), although the abrupt change of the *T*_mean_ in April was insignificant (Figure 5i). These observations suggested that temperature abrupt changes were followed by the phenological abrupt changes of cotton bollworm, except for *t*_1_. Additionally, climate change affected the phenologies of cotton bollworm and the eclosion period because the days between *t*_1_ and *t*_5_ determined the eclosion period. The abrupt change of abundance was later than that of *T*_mean_ in April and was coinstantaneous with *T*_mean_ in March and the date of *t*_2_. The *T*_mean_ in April may produce a greater effect on the abrupt change of abundance. The abrupt change of the eclosion period was in 1997 and was coinstantaneous with *T*_mean_ in April. For a 1°C increase in *T*_mean_ in April, the date of *t*_1_ advanced by 3.637 days (Table 2). Furthermore, the explained variance of *t*_1_ to the eclosion period was 57.8%; thus, the eclosion period was mainly affected by the *T*_mean_ in April, and they had same abrupt change trends (Figure 5i,k).

The abrupt change year of wheat yield was in 1998, but this change was not significant (Figure 5a). Meanwhile, the abrupt change year of anthesis was in 1991 (Figure 5g). Yield increased with the advance of anthesis (Figure 2a); however, the abrupt changes of yield and anthesis were not coinstantaneous. Contrarily, the abrupt changes of yield and *T*_mean_ in March and the dates of *t*_1_, *t*_2_ and *t*_5_ were almost coinstantaneous. This showed that wheat yield and *T*_mean_ in March had same change trends (Figure 5a,h) because wheat was in the stage from booting to heading in March, and the *T*_mean_ in March had significant effects on wheat yield (Figure 2c).

### 3.4 The proportion of explained variance of the affecting factors

As multicollinearity existed between the variables, the proportions of explained variance of the affecting factors were analyzed by PLS (Table 3). The factors with the greatest effects on the appearance dates of *t*_1_–*t*_5_, abundance, wheat yield, eclosion days, and wheat anthesis were AT_1_, *T*_max_ in March, *T*_max_ in April, minimum air temperature (*T*_min_) in March, precipitation in winter, AT during the eclosion period, abundance, AT during the eclosion period, and *T*_max_ in December, respectively, and the proportion of explained variance was 70.0%, 55.0%, 40.1%, 48.2%, 38.5%, 70.6%, 69.2%, 87.3%, and 32.3%, respectively (Table 3). AT had the greatest impact on *t*_1_; however, AT had only the second greatest impact on *t*_2_–*t*_5_, and the proportion of explained variance varied from 21.0% to 37.0%, which was far lower than the proportion of 70% on *t*_1_. The precipitation in winter and the AT had almost identical impacts on the date of *t*_5_; however, the impacts of AT on *t*_2_–*t*_4_ were markedly lower than those of Factor 1 on *t*_2_–*t*_4_ (Table 3). This might suggest that the AT determined the beginning of the eclosion period and that the effects of AT on the dates of *t*_2_–*t*_5_ decreased. Meanwhile, the *T*_mean_ in March and April also had greater effects on *t*_1_–*t*_5_ (Table 3).

**Table 3:** The proportion of variance explained of affecting factors to appearance dates of *t*_1_–*t*_5_, abundance, eclosion days, wheat anthesis, and yield from 1990 to 2007

	Factor 1 (%)	Factor 2 (%)	Factor 3 (%)	Factor 4 (%)	Factor 5 (%)
*t*_1_	AT_1_ (70.4)	*A* (18.8)	*B* (4.4)	*E* (3.4)	*D* (1.7)
*t*_2_	*B* (55.0)	AT_2_ (21.0)	*A* (14.7)	*E* (5.8)	*D* (1.6)
*t*_3_	*D* (40.1)	AT_3_ (31.3)	*A* (7.2)	*C* (8.7)	*B* (2.6)
*t*_4_	*C* (48.2)	AT_4_ (29.6)	*A* (6.6)	*E* (4.0)	*F* (0.6)
*t*_5_	*G* (38.5)	AT_5_ (37.0)	*A* (1.7)	*H* (5.7)	*I* (3.7)
Abundance	AT_t_ (70.6)	*J* (14.3)	*K* (6.8)	*L* (2.6)	*M* (2.3)
Yield	Abundance (69.2)	Anthesis (18.0)	*A* (3.8)	Date of *t*_1_ (0.7)	AT_t_ (0.7)
Eclosion days	AT_t_ (87.3)	Date of *t*_1_ (7.3)	AT_1_ (3.0)	Abundance (0.6)	*N* (0.7)
Anthesis	*H* (32.3)	*O* (19.7)	*P* (14.5)	*Q* (12.0)	*R* (7.4)

AT_*n*_: accumulated temperature from January 1 to the date of *t*_*n*_; AT_t_: accumulated temperature during the eclosion period; *A*:* T*_mean_ in March; *B*:* T*_max_ in March; *C*:* T*_min_ in March; *D*:* T*_mean_ in April; *E*:* T*_max_ in April; *F*: precipitation in February; *G*: precipitation in winter; *H*:* T*_max_ in December; *I*:* T*_max_ in January; *J*: precipitation in January; *K*:* T*_min_ in February; *L*:* T*_mean_ on date of *t*_1_; *M*: the eclosion period; *N*: the days between *t*_1_ and *t*_2_; *O*: temperature on the date of *t*_5_; *P*: precipitation in March; *Q*:* T*_mean_ from *t*_4_ to *t*_5_; *R*: precipitation in December. The values in brackets were the proportion variance explained of affecting factors.

Accumulated temperature during the eclosion period with an explained variance of 70.6% became the factor with the greatest effect on abundance (Table 3). This shows that the overwintering pupae need enough AT for eclosion. Precipitation in January and minimum air temperature (*T*_min_) in February had explained variances of 14.3% and 6.8%, respectively, and they became the factors with the second and third greatest effects on abundance, both decreasing abundance. *T*_mean_ on the date of *t*_1_ and the eclosion period increased abundance, but they had very low explained variance (Table 3).

Abundance had the greatest impact on wheat yield with an explained variance of 69.2% (Table 3). Furthermore, the anthesis with an explained variance of 18.0% became the second greatest affecting factor (Table 3) because the advance of anthesis increased yield (Figure 2a). However, the date of *t*_1_ only had an explained variance of 0.7% for wheat yield (Table 3), although the relationship between yield and the date of *t*_1_ had a significant negative correlation (Figure 2b).

AT during the eclosion period with an explained variance of 87.3% became the major affecting factor for eclosion days (Table 3), and the AT before the date of *t*_1_ only had an explained variance of 3%. This suggested that the AT during the eclosion period was the most important affecting factor. An advance of the date of *t*_1_ prolonged the eclosion period and increased AT during the eclosion period, which would create more pupae eclosion.

## 4 Discussion

### 4.1 Effects of climate change on the phenology of cotton bollworm

Climate change has caused climate abruption. Climate abrupt change is a change from one stable status to another stable status and is characterized by dramatic changes in climate that may vary spatiotemporally from one statistical characteristic to another statistical characteristic (Fu & Wang, 1992). Phenology abrupt change usually appears after temperature abrupt change (Liu, Gong, et al., 2007; Liu, Zheng, et al., 2007); however, the abrupt change of wheat anthesis was earlier than that of *T*_mean_ in March and April (Figure 5b,g,h, i). This might suggest that other factors could also affect phenology abruption. The abrupt change of wheat yield was in 1998 and was later than the abrupt change of *T*_mean_ in April and earlier than the abrupt change of *T*_mean_ in March. However, all phenological abrupt changes were around the abrupt change of *T*_mean_ in March and April. In the other hand, these results also showed that the responses of phenology to climate change were sensitive, and this might be used as an index of evaluating climate change.

Climate warming advanced the dates of *t*_1_–*t*_5_ and prolonged the eclosion period by 1.277, 0.788, 0.382, 0.420, 0.193, and 1.090 days, respectively (Table 1), while the results of Ouyang et al. (2016) were 0.4512, 0.2484, 0.3167, 0.1115, 0.1838, and 0.7249 days, respectively. Markedly, the results of Ouyang et al. (2016) were lower than those in this study. This might be because the geotypes of cotton bollworm were different. The *H. armigera* populations in China can be classified into four regional categories, the tropical, subtropical, temperate, and XinJiang geotypes, with respect to differences in diapause, cold hardiness, and genetic variation, and different geotypes are highly adapted to local environments (Wu & Guo, 1997). The geotype of cotton bollworm in Ouyang et al. (2016) was a temperature geotype, and in this study, the geotype was Xinjiang. In addition, the population of the Xinjiang geotype has a higher cold tolerance than do those in other regions (Wu & Guo, 2000; Wu, Guo, Wei, & Sun, 1997). In addition, the characteristic of high adaption to the local environment might be another reason for observed differences because the distance between the two sites was approximately 3,000 km, and there were different climate environments. As Parmesan (2007) stated, the numerous phenological responses to climate warming varied among the different regions and taxonomic groups. Thus, when the effects of climate change on phenologies of cotton bollworm are studied, the different geotype populations should be considered.

Precipitation delayed the appearance of overwintering pupae (Huang & Li, 2015; Ouyang et al., 2016). The amount and timing of precipitation affect pest abundance, and the predictions of trap catches for *H. armigera* are based on previous catches and precipitation (Maelzer, Zalucki, & Laughlin, 1996). Rain plays different roles in different months; for example, rainfall in September–October is more important than that in September for *Helicoverpa punctigera* (Maelzer et al., 1996) and vice versa for *H. armigera* (Maelzer & Zalucki, 1999). Rain increases soil moisture, which is helpful for wheat growth, and the effect of rain on the *H. armigera* population is substantial (Maelzer & Zalucki, 1999). There were no adverse impacts of extreme temperatures and humidity on the eggs and first‐instar survival weekly means on population changes in light trap catches (Maelzer & Zalucki, 1999), but this can reduce the abundance of *H. armigera*. In this study, the precipitation from December of last year to April of this year, with a mean value of 15.04 mm, varied from 0 to 58.7 mm during the period of 1989–2007. Such little precipitation produced few effects on eclosion via PLS analysis in this study. The sites used in this study and in that of Maelzer et al. (1996) and Maelzer and Zalucki (1999) had different environmental conditions, so the major affecting factors were different.

Climate warming advanced the phenologies of cotton bollworm and wheat anthesis and prolonged the eclosion period. Thus, the increased AT increased the eclosion ratio of the overwintering pupae (Huang & Li, 2015), which can produce substantial wheat damage. The spring phenology of cotton bollworm had significant correlations with *T*_mean_ in March and April (Table 2). This hinted that *T*_mean_ in March and April had considerable effects on cotton bollworm, and the phenological outset had significant correlations with the temperatures of 2–3 months before the outset of the phenology. This provides an opportunity to increase prediction veracity.

### 4.2 Effects of climate change on the match/mismatch for the phenology of cotton bollworm and wheat anthesis

Increasing adult abundance of overwintering *H. armigera* worsened wheat damage (Ouyang et al., 2016). The increase in abundance may be due to global warming (Kiritanil, 2006), which was supported by this study. However, this study also suggested that there was enough food for more pupae to become adults.

The eclosion ratios of *t*_2_–*t*_4_ are 68.3% eclosion (Ouyang et al., 2016), so this stage was regarded as a key stage in the study. The advance of *t*_2_ increased abundance (Figure 4d), and the advances of *t*_3_ and *t*_4_ may increase abundance, although they were insignificantly correlated (data not shown). Similarly, the increase in AT, days, and mean temperature between *t*_2_ and *t*_3_, *t*_3_ and *t*_4_, and eclosion period may also increase abundance (Figure 4f,h,i,l), although some of the relationships were insignificant (data not shown). At this research site, wheat anthesis usually appeared in the first 10 days in May, and the eclosion period of the overwintering pupae usually occurred from the second 10 days in April to the third 10 days in May. If the phenologies of cotton bollworm and wheat anthesis were mismatched, the larva of cotton bollworm could face a scarcity of food, and adult abundance would decline. The advance of wheat anthesis would likely produce more adults (Figure 3d); however, the increase in *T*_max_ in December delayed wheat anthesis (Figure 3e). With climate warming, this might lead to a phenology mismatch that would be harmful to cotton bollworm larvae in the future.

As the ova of the first generation of *H. armigera* are mainly kept in the plant ear (He, Wang, & Yang, 1996), and *H. armigera* feeds on multifarious foods from the third instar (Ge et al., 2005), even the larva can feed from heading to ripening (Ouyang et al., 2016). This leads to a wheat yield loss of 5% (Dong et al., 1993) and a total biomass loss of approximately 40% (Ouyang et al., 2016). However, larva in the spring might suffer a scarcity of diet (Ge et al., 2005; Reddy et al., 2015) because climate warming advanced the phenologies of wheat (Wang et al., 2008) and *H. armigera* (Huang & Li, 2015; Ouyang et al., 2016), and wheat anthesis likely had a different response than did the pupae to climate warming. In this study, the trend changes of the dates of *t*_1_–*t*_4_ and the eclosion days were faster than those of wheat anthesis (Table 1), and the only trend change that was lower than that of wheat anthesis was the date of *t*_5_ (Table 1). Furthermore, for a 1‐day advance of *t*_1_–*t*_5_ of *H. armigera*, wheat anthesis advanced by 0.020, 0.017, −0.011, 0.043, and 0.129 days, respectively, although the correlations were insignificant (*p *>* *.05). In addition, when the date of *t*_3_ advanced, the wheat anthesis appeared to have a slight delay. This also illustrated that the change in wheat anthesis was asynchronous with that of *H. armigera*. As asynchronous responses to climate warming, the gaps between phenologies of *H. armigera* and wheat anthesis were likely to be expanded. Thus, the larva of the first‐generation cotton bollworm was likely to face more scarcities of diet and an increase in the risk of death. The phenology advances of *t*_1_–*t*_5_ increased wheat yield (Figure 2b–f), and this hinted that the earlier the appearance of larva in the spring, the greater possibility that the larva would die from a scarcity of diet. In summation, the mismatch of phenology increased wheat yield. In addition, the gaps between the phenologies of *t*_1_–*t*_5_ and wheat anthesis were (mean ± *SD*) −8.78 ± 10.77, 1.17 ± 8.35, 10.50 ± 6.64, 16.56 ± 6.43, and 22.50 ± 5.32 days (Figure 1d), respectively. This showed that the mean appearance dates of *t*_2_–*t*_5_ were later than the date of wheat anthesis, and the appearance date of *t*_1_ was the only date that was earlier than the date of wheat anthesis. However, gap_1_ had the largest *SD* of 10.77 days and gap_5_ had the smallest *SD* of 5.32 days because the date of *t*_1_ had the largest variation range and the trend change of *t*_1_ was faster than that of wheat anthesis (Table 1). The date of *t*_5_ had the smallest variation range, although the trend change rate of *t*_5_ was faster than that of wheat anthesis (Table 1). The *SD*s of gap_1–5_ gradually decreased, showing that the variation ranges of gap_1–5_ between wheat anthesis and the phenology of *H. armigera* were gradually reduced with climate warming, and the later the phenology change was, the smaller the impact from climate was. While the trends over time of gap_1–5_ were −0.943, −0.455, −0.049, −0.087, and 0.148 days/year, respectively, only the relationship between gap_1_ and year was significant (*Y* = −0.943*X* + 0.183, *R* ^2^ = .218, *p *=* *.050). This illustrated that gap_1–5_ showed expanded trends, viz., and interval days between wheat anthesis and the phenology of *H. armigera* were increasing. Gap_1–4_ showed advanced trends, and the gap_1–4_ increased by 0.943, 0.455, 0.049, and 0.087 days per year, respectively, and the gap_5_ showed a delayed trend and was delayed by 0.148 days per year. This suggested that the asynchronous responses in the relative growth rate of wheat and overwintering *H. armigera* were expanded. Thus, with the increase in gap_1–4_ in the context of climate warming, the larva of *H. armigera* in the spring is likely to face more food scarcity, and mortality may increase.

Although the advance of the spring phenology of the overwintering generation of *H. armigera* could increase the wheat yield, the explained variance values of the phenologies were too low. The date of *t*_1_ had an explained variance of 0.7% for wheat yield, and the impacts of dates of *t*_2_–*t*_5_ were excluded from the five major affecting factors (Table 3). The factor most affecting wheat yield was the abundance of the overwintering generation, which explained 69.2% of the variance in wheat yield (Table 3). Thus, the abundance of the overwintering generation might be the major research target for wheat yield. As mentioned above, the abundance might increase because of an increase in AT, the prolongation of the eclosion period, and climate warming (Table 3). Therefore, the increase in abundance likely reduced wheat yield. This was consistent with the conclusions of Ouyang et al. (2016).

Different trophic levels cannot shift their phenologies at the same rate (Durant et al., 2007), and *H. armigera* and wheat were not exceptions. Reddy et al. (2015) presumed that AT during the winter caused the mismatch between *H. armigera* and wheat, and they suggested that AT should be given more attention as a factor in phenological events. In this study, as mentioned above, the AT had a substantial effect on *t*_1_–*t*_5_ and the eclosion days (Table 3), but AT had little effect on wheat anthesis; contrarily, *T*_max_ in December was the greatest affecting factor (Table 3). Furthermore, the relationships between AT and air temperature in the winter and wheat anthesis were insignificant (*p *>* *.05). The results in this study are partly supported by Reddy et al. (2015).

Many factors could lead to the death of cotton bollworm. Increasing temperature may cause plants to produce less nutritional quality tissue for herbivores, which may increase herbivore consumption requirements and lead to increased risk of plant consumption (Emmerson et al., 2004). Different hosts can cause diapausing pupae to produce significantly different supercooling points (Ouyang et al., 2011), and a decrease in the cold hardiness of *H. armigera* can lead to an increase in mortality (Wu & Guo, 1997). Moreover, transgenic crops can kill *H. armigera* and increase cotton yields and reduce pesticide spraying (Pray, Huang, Hu, & Rozelle, 2002; Pray, Ma, Huang, & Qiao, 2001; Wu et al., 2008); however, pest resistance to transgenic crops is increasing and will likely reduce the impact of transgenic crops (Tabashnik, 1994). Refuge crops can delay moth physiological resistance to transgenic crops (Andow & Ives, 2002; Gould, 1998; Ives & Andow, 2002; Ives, Glaum, Ziebarth, & Andow, 2011). Suitable refuge crops are determined by weather and local agricultural practices and may include a combination of crops (Lu, Zalucki, Perkins, Wang, & Wu, 2013). Wheat is likely to be used as a refuge crop in northwestern China (Lu et al., 2013), and this would result in more damage to wheat yield caused by bollworms. On the other hand, *H. armigera* trap‐catch change is affected by wind speed, temperature, night length, and moonlight (Morton, Tuart, & Wardhaugh, 1981). These factors should be considered in future studies.

## 5 Conclusion

Warmer temperatures in the spring have advanced the phenologies of cotton bollworm and wheat anthesis, and the phenology changes in overwintering *H. armigera* were faster than that in wheat anthesis. This extended the eclosion period of pupae and increased the adult abundance of the overwintering generation. In addition, more larvae were recruited in the first generation and consequently damaged the wheat. The abrupt changes of the phenologies of cotton bollworm, wheat anthesis, and climate were asynchronous, but the abrupt changes of the phenologies were after or around the abrupt change of climate. In addition, the asynchronous responses in change rates of wheat and overwintering *H. armigera* were expanded and could decrease wheat yield due to climate warming in the future. AT was the major factor affecting *t*_1_, abundance, and eclosion days, while temperature in March and April and precipitation in the winter mainly affected *t*_2_, *t*_3_ and *t*_4_, respectively.

## Conflict of Interest

The authors have no conflict of interests to declare.
